# N-Terminal Pro-B-Type Natriuretic Peptide and Cardiac Troponin T in Stable Renal Transplant Recipients and All-Cause Mortality, Cardiovascular, and Renal Events

**DOI:** 10.3390/biom15091298

**Published:** 2025-09-09

**Authors:** Zbigniew Heleniak, Marcel G. Naik, Georgios Eleftheriadis, Tomasz Madej, Fabian Halleck, Alicja Dębska-Ślizień, Klemens Budde

**Affiliations:** 1 Department of Nephrology, Transplantology and Internal Medicine, Medical University of Gdansk, 80-210 Gdańsk, Poland; adeb@gumed.edu.pl; 2 Medizinische Klinik mit Schwerpunkt Nephrologie und Internistische Intensivmedizin, Charité–Universitätsmedizin Berlin, 10117 Berlin, Germany; marcel.naik@charite.de (M.G.N.); georgios.eleftheriadis@charite.de (G.E.); fabian.halleck@charite.de (F.H.); klemens.budde@charite.de (K.B.); 3 Department of Population Health Monitoring and Analysis, National Institute of Public Health NIH-National Research Institute, 24 Chocimska Street, 00-791 Warsaw, Poland; tomasz.madej94@gmail.com

**Keywords:** all-cause mortality, biomarker, cardiovascular disease, kidney transplantation

## Abstract

Introduction: In renal transplant recipients (RTRs), kidney graft failure and cardiovascular (CV) disease are prevalent and associated with mortality. Objectives: The objective of the study was to evaluate biomarkers, (cardiac troponin T (cTnT) and N-terminal pro-B-type natriuretic peptide (NT-proBNP)), to identify RTRs who are at greater risk of death, CV event, and graft renal survival. Patients and methods: A total of 342 stable RTRs were enrolled in this study, with a median follow-up time of 54 months. The probability of death, CV event, and renal graft survival were calculated using Kaplan–Meier analysis for the group defined by cTnT and NT-proBNP levels above the cutoff values. Results: The probability of death for troponin T level above the cut-off was 23% and for NT-proBNP 29%. For CV events the probability for troponin T was 20% and for NT-proBNP it was 21%. Troponin T concentrations above the cutoff point suggested a 25% probability of death-censored graft survival. For NT-proBNP, it was 26%. The probability of overall graft survival was 38% for patients with higher troponin T levels, and 40% for NT proBNP. Conclusions: These data suggest that cTnT and NT-proBNP could potentially identify patients at high risk for death, CV event, and graft survival.

## 1. Introduction

While long-term graft survival has not changed significantly, improvements in immunosuppressive regimens have enhanced short-term graft survival [1]. However, graft failure within 5 years occurs in 1 out of 5 renal transplant recipients (RTRs) [2,3,4]. In addition, cardiovascular disease (CVD) is highly prevalent in patients after kidney transplantation and significantly affects morbidity and death [5,6]. In addition to the standard risk factors (diabetes, obesity, hyperlipidemia, age), cardiovascular (CV) complications in RTRs are also linked to nontraditional factors unique to this population, including renal function, proteinuria, immunosuppressive therapy, length of dialysis therapy, inflammation, anemia, and elevated arterial stiffness [7,8,9]. The use of biomarkers to identify RTRs who are more likely to experience unfavorable outcomes could improve patient care for these individuals. The assessment of cardiac troponin I (cTnI) and T (cTnT) has the potential to predict adverse outcomes and is used to diagnose acute myocardial infarction and coronary ischemia [10,11,12]. However, elevations in cardiac troponins have been reported in several other acute and chronic conditions and are not exclusive to acute myocardial infarction [13]. Elevated levels of cardiac troponin have been reported in patients with chronic kidney disease (CKD). This may restrict the use of cTnT or cTnI assays for diagnosing acute myocardial events in patients with reduced glomerular filtration rate (GFR), but it may also highlight the predictive value of troponins for CV complications in CKD patients [14,15]. B-type natriuretic peptide (BNP) and N-terminal pro-B-type natriuretic peptide (NT-proBNP) have emerged as established biomarkers for heart failure (HF) [12]. Since BNP and NT-proBNP are responsive to volume overload, their levels are elevated in CKD [16]. Jarolim et al. showed that baseline BNP concentrations were associated with all-cause mortality, CV, and renal events in stable RTRs receiving folic acid and vitamin B supplementation [17]. Furthermore, BNP was more strongly linked to adverse outcomes in this population than cTnI, which had a stronger link to all-cause mortality. Conversely, an increase in cTnT within 3 weeks to a year following kidney transplantation was associated with worse patient survival rates, and a subset of patients at high risk for death or CV events was identified by the failure of cTnT levels to normalize posttransplant [18]. The objective of the study was to further evaluate the predictive value of NT-proBNP and cTnT for important clinical outcomes in RTRs. The primary endpoint was all-cause mortality, while the secondary endpoints included adverse CV and renal events, overall graft survival, and death-censored graft survival.

## 2. Materials and Methods

This study enrolled 342 stable RTRs, who were transplanted between 1994 and 2018 and attended the outpatient unit of the Department of Nephrology at Charité-Universitätsmedizin Berlin, Germany, between February and July 2018. All patients provided written informed consent, and the study was conducted in accordance with the Declaration of Helsinki. The study was approved by the local ethics committee (EA 1/252/17).

Inclusion criteria included all patients transplanted between 1994 and 2018, who signed the consent of participation in the study and there were all data regarding the death, CV event and graft survival in the follow-up period of time. Exclusion criteria were defined as a lack of consent for this study and no data regarding the follow-up in terms of death, CV event and graft survival.

We analyzed demographic data, immunosuppression status, and available clinical information, including transplant characteristics, hypertension, diabetes, and CVD, as previously described [19]. Data on laboratory test results, such as serum creatinine, estimated glomerular filtration rate (eGFR) calculated using the CKD-EPI equation, cTnT, and NT-proBNP levels, were obtained from the medical records at the start of the observation period. Increased serum level of creatinine was defined as concentration >1.2 mg/dL (>106.1 µmol/L) and the reference range for eGFR was described as ≥60 mL/min/1.73 m^2^. Abnormalities in serum levels of cTnT and NT-proBNP were defined as concentrations >14 ng/L and >125 ng/L, respectively.

### 2.1. Definition of Outcomes

The primary endpoint of the study was all-cause mortality. Secondary endpoints included renal and CV events. Renal events were defined as a return to dialysis or an increase of at least 30% in serum creatinine, while CV events were characterized by incidents of stroke, myocardial infarction, or severe peripheral artery disease.

Moreover, overall graft survival was assessed, with graft loss defined as a return to dialysis or death with a functioning graft. Death-censored graft survival was also evaluated, where graft loss was defined as a return to dialysis, with death treated as a censoring event.

### 2.2. Follow-Up

The participants were followed up through the hospital system (T-Base) in February 2023, and the occurrence of death, CV, and renal events during this observation period was recorded [20].

### 2.3. Statistical Analyses

Due to the presence of extreme values in the variables determining cTnT (ng/L) and NT-proBNP (ng/L) levels, winsorization was applied [21]. Extreme observations that significantly deviated from the overall distributions could have biased the analysis and potentially led to incorrect generalizations of the conclusions. Asymmetric winsorization was applied to the right tail of the distribution. The 95th percentile was used as the boundary, which helped balance the influence of outliers while maintaining the integrity of the data. Before winsorization, the maximum value, skewness, and kurtosis for cTnT were 183 ng/L, 3.77, and 21.5, respectively; after winsorization, these values were 48.95 ng/L, 1.29, and 0.73. For NT-proBNP, the baseline values were 51,672 ng/L, 10.55, and 133.19, respectively; after processing, the values were 3376 ng/L, 1.91, and 2.44.

Descriptive statistics of the analyzed cohort are presented for the entire group and by quartiles of cTnT and NT-proBNP concentrations. Patient characteristics were compared using descriptive statistics: means and standard deviations for continuous variables, and counts and frequencies for categorical variables. Differences in cohort characteristics were tested using the one-way ANOVA and the Kruskal–Wallis rank-sum test for continuous variables with normal and nonnormal distributions, respectively. For categorical variables, Pearson’s chi-squared test and Fisher’s exact test were used for factorial variables with counts greater than or equal to 5, and less than 5, respectively. The Kaplan–Meier model and log-rank test were used to estimate probabilities and test for differences in outcomes based on the stratified cohort using the cutoff values.

We analyzed time-to-event outcomes using Cox proportional hazards generalized additive models (Cox-GAMs) with penalized thin-plate regression splines to accommodate nonlinear effects. The primary exposures were high-sensitivity Troponin T (cTnT) and NT-proBNP.

#### 2.3.1. Thin-Plate Spline Explanation

To allow relationships to be curved rather than forced to be straight lines, we used thin-plate regression splines (via mgcv package, Wood, 2017 ). These fits produce smooth curves and the software automatically limits how “wiggly” they can be so the model captures broad patterns without chasing noise. We allowed moderate flexibility (*k* = 10 for cTnT and NT-proBNP; *k* = 4 for other continuous variables) and confirmed this was sufficient with routine diagnostics. When a curve was unnecessary, the method effectively shrank it toward a straight line.

#### 2.3.2. Winsorisation Explanation

To limit undue influence of extreme biomarker values while retaining all observations, we applied one-sided winsorization: hs-cTnT and NT-proBNP values above the 95th percentile were set equal to the 95th percentile (no lower-tail capping). This caps only the upper tail and does not remove observations or change rank ordering except among the capped values.

Prespecified covariates were: age (spline), sex, time since kidney transplantation (spline), preemptive kidney transplantation (KTx) status, eGFR (spline), current use of tacrolimus, cyclosporine, and corticosteroids, and history of coronary artery disease (CAD). These variables were selected based on the recommendations of a panel of clinical experts in transplantation medicine, reflecting factors with established or plausible associations with post-transplant outcomes and potential confounding effects on the studied relationships.

For each primary exposure and outcome, we identified a data-driven cutoff as the value where the model’s smooth for that exposure crossed the risk-neutral line (HR = 1). If no crossing was present in this range, we selected the value closest to neutrality. The resulting cutoff defined the below (<cutoff) and above (≥cutoff) groups and the exposure intervals summarized in the tables.

Effect summaries. We reported two quantities:

• Standardized point HR (population-standardized, point-specific): the hazard ratio predicted if everyone were set to a specific exposure value (e.g., quartile cut-point or model-derived cutoff), averaged over the cohort’s covariate distribution.
• Subgroup-average HR (interval) (as-observed): the average of model-predicted hazard ratios among participants whose observed exposure falls within a given interval (e.g., below vs. above the cutoff; quartiles).

This separation makes clear that standardized point HRs answer “What would the average HR be if everyone had this exposure value?”, while subgroup-average HRs answer “What is the average HR among people who actually had exposure in this interval?”

To display the functional form and risk-neutral point, we plotted each exposure’s partial effect on the hazard scale with 95% pointwise confidence bands, adding a horizontal line at HR = 1 and a vertical line at the chosen cut-point. Using these cut-points, we plotted Kaplan–Meier survival curves for Above vs. Below and compared groups with the log-rank test (unadjusted). Discriminative performance of each biomarker cut-point was assessed at 12, 24, 36, and 48 months using time-dependent ROC curves with inverse probability of censoring weighting (IPCW; marginal method). The binary above vs. below variable served as the marker, and AUCs with 95% confidence intervals were reported for each outcome and time point.

The proportional hazards assumption was assessed with scaled score residuals for parametric terms and visual inspection for smooth effects. Concurvity among spline terms was examined; no values exceeded 0.4 in the worst case. A two-sided *p* < 0.05 defined statistical significance. Because this was an exploratory pilot investigation, no a priori sample-size or power calculation was performed.

Analyses were performed in the R environment (version 4.1.3; R Core Team, R Foundation for Statistical Computing, Vienna, Austria) using RStudio Desktop (Posit Software, PBC, Boston, MA, USA) and the mgcv package (Wood, 2017) for Cox-GAM fitting, marginaleffects for standardized and subgroup averages, and time ROC for time-dependent ROC analyses.

## 3. Results

### 3.1. Study Population Characteristics

Table 1 and Table 2 present the characteristics of the study cohort based on the quartiles of cTnT and NT-proBNP levels in the blood at the start of the observation period. At baseline, the mean time since kidney transplantation was 93 months, with the shortest and longest times being 1 and 384 months, respectively. The mean creatinine value was 1.67 mg/dL, and the mean eGFR was 50 mL/min/1.73 m^2^. Significant differences in kidney graft function were observed across the four quartile groups for both biomarkers. The prevalence of coronary artery disease (CAD), HF, and diabetes was higher among patients with higher values of cTnT and NT-proBNP.

There were differences in the immunosuppression regimen (tacrolimus, steroids) across in both biomarker groups. Higher levels of troponin T and NT-proBNP were associated with older age. The duration of renal replacement therapy (RRT) before kidney transplantation was longer in patients in the third and fourth quartiles of biomarker levels compared to those with lower levels of cTnT and NT-proBNP. It is worth noting that the majority of RTRs who underwent pre-emptive kidney transplantation were in the first quartile for both biomarker levels.

Patients with higher troponin T concentrations were more likely to be male and to have more CV events at the start of the observation. However, higher NT-proBNP concentrations were associated with a higher proportion of women relative to men.

The mean and median follow-up times were 48.4 and 54 months, respectively. The minimum and maximum observation periods were 5 and 59 months, respectively. The 4.9-year probabilities of occurrence, calculated using Kaplan–Meier analysis, were as follows: death 10% (95% CI: 6%; 13%); CV event 10% (95% CI: 5%; 14%); renal event 45% (95% CI: 34%; 54%); graft failure or death (overall graft survival) 17% (95% CI: 13%; 21%); and graft failure (death-censored graft survival) 11% (95% CI: 8%; 15%).

Next, we developed Cox models for both biomarkers to predict death, CV events, and renal events, adjusted for age (spline), sex, time since kidney transplantation (spline), preemptive kidney trans-plantation (KTx) status, eGFR (spline), current use of tacrolimus, cyclosporine, and corticosteroids, and history of coronary artery disease (CAD). (Table 3).

### 3.2. Death

The nonlinear Cox–GAM identified a neutral troponin T concentration of ≈12.25 ng/L. Below this threshold, the conditional (as-observed) average HR was 0.19 (81% lower; 95% CI 0.05–0.74; *p* = 0.017). Above the cutoff, the conditional average HR was ≈6- to 16-fold higher (HR = 6.98; 95% CI 1.70–28.72; *p* = 0.007; highest bin HR = 15.98; 95% CI 3.45–74.04; *p* < 0.001). Standardized point HRs rose steeply across the upper range (e.g., upper-quartile HR = 7.74; 95% CI 1.56–38.41; *p* = 0.012).

**Figure 1:**
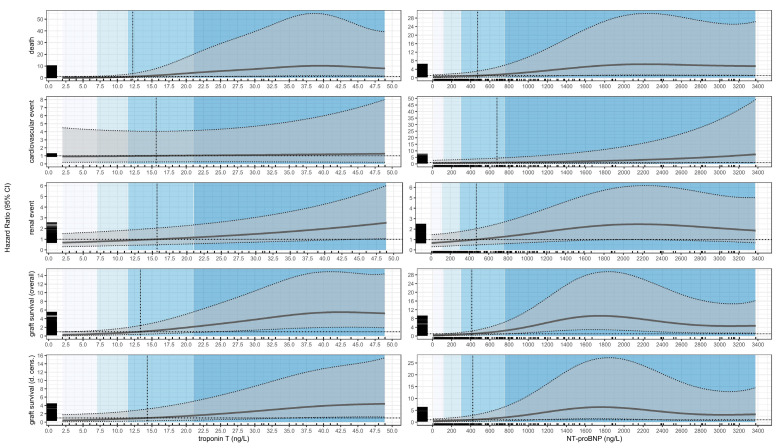
Nonlinear estimates from adjusted Cox models with 95% confidence intervals, distribution of observations, and gradational backgrounds indicating quartiles of troponin T and NT-proBNP concentrations. Grey bold line: point estimates; grey ribbon: 95% confidence intervals.

For NT-proBNP, the estimated cutoff was ≈471.53 ng/L. Below this value, the conditional average HR was 0.56 (44% lower; 95% CI 0.17–1.79; *p* = 0.30), whereas above the cutoff it was ≈8- to 11-fold higher (HR = 8.26; 95% CI 2.51–27.22; *p* < 0.001; top bin HR = 11.45; 95% CI 3.24–40.48; *p* < 0.001). Standardized point HRs at extreme concentrations were similarly elevated (e.g., upper-quartile HR = 7.43; 95% CI 1.54–35.83; *p* = 0.013) (Figure 1).

### 3.3. Cardiovascular Events

For troponin T, the cutoff was ≈15.63 ng/L. Below this point, the conditional average HR was near null (HR = 0.91; *p* = 0.9). Above it, the average HR was ≈3.3-fold higher but imprecise (HR = 3.30; 95% CI 0.69–15.69; *p* = 0.13). Standardized point HRs across quartiles remained near 1.

For NT-proBNP, the cutoff was ≈673.94 ng/L. Below the cutoff, the conditional average HR was null (HR = 1.03; *p* > 0.9). Above it, the average HR was ≈5.2-fold higher (HR = 5.24; 95% CI 1.03–26.77; *p* = 0.046). The standardized point HR at the highest concentration was large (HR = 11.68; 95% CI 1.73–78.93; *p* = 0.012) (Figure 1).

### 3.4. Renal Events

For troponin T, the cutoff was ≈15.68 ng/L. Below the cutoff, the conditional average HR was ≈2.5-fold higher (HR = 2.49; 95% CI 1.23–5.02; *p* = 0.011). Above it, the average HR was ≈10- to 12-fold higher (HR = 10.13; 95% CI 4.66–22.04; *p* < 0.001; higher bin HR = 12.49; 95% CI 5.65–27.60; *p* < 0.001). Standardized point HRs increased monotonically across quartiles (e.g., upper-quartile HR = 10.21; 95% CI 4.31–24.17; *p* < 0.001) (Figure 1).

For NT-proBNP, the cutoff was ≈298.50 ng/L. Subgroup-average HRs were ≈2.3-fold higher below the cutoff (HR = 2.27; 95% CI 1.12–4.57; *p* = 0.022) and ≈8.3- to 15.1-fold higher above it (HR = 8.29; 95% CI 3.96–17.35; *p* < 0.001; top bin HR = 15.12; 95% CI 6.72–34.01; *p* < 0.001). Standardized point HRs likewise rose with concentration (e.g., 753.5 ng/L HR = 5.49; 95% CI 2.61–11.54; *p* < 0.001) (Figure 1).

### 3.5. Graft Loss (Overall Survival)

For troponin T, the cutoff was ≈13.37 ng/L. Below the cutoff, the conditional average HR was 0.47 (53% lower; 95% CI 0.19–1.14; *p* = 0.10). Above it, the average HR was ≈5.9- to 11.1-fold higher (HR = 5.85; 95% CI 2.35–14.56; *p* < 0.001; higher bin HR = 11.12; 95% CI 4.25–29.09; *p* < 0.001). Standardized point HRs were likewise elevated at higher concentrations (e.g., at 48.95 ng/L, HR = 6.92; 95% CI 2.52–19.00; *p* < 0.001) (Figure 1).

For NT-proBNP, the cutoff was ≈410.81 ng/L. Below the cutoff, subgroup-average HRs were near-null to slightly lower (HR = 0.66; 95% CI 0.29–1.55; *p* = 0.3). Above it, the average HR was ≈11- to 20-fold higher (HR = 11.18; 95% CI 4.54–27.51; *p* < 0.001; top bin HR = 19.61; 95% CI 7.36–52.25; *p* < 0.001). Standardized point HRs again climbed sharply (e.g., 753.5 ng/L HR = 4.66; 95% CI 1.80–12.10; *p* = 0.002) (Figure 1).

### 3.6. Graft Loss (Death-Censored)

For troponin T, the cutoff was ≈14.36 ng/L. Subgroup-average HRs were null below the cutoff (HR = 0.96; *p* > 0.9) and ≈7.6- to 13.9-fold higher above it (HR = 7.64; 95% CI 2.36–24.72; *p* < 0.001; higher bin HR = 13.87; 95% CI 4.13–46.55; *p* < 0.001). Standardized point HRs at higher concentrations were similarly increased (e.g., 48.95 ng/L HR = 9.46; 95% CI 2.70–33.13; *p* < 0.001) (Figure 1).

For NT-proBNP, the cutoff was ≈420.93 ng/L. Below the cutoff, subgroup-average HRs were near-null (HR = 1.17; *p* = 0.8). Above it, the average HR was ≈17-fold higher (HR = 16.94; 95% CI 5.35–53.68; *p* < 0.001). Standardized point HRs again rose across the upper range (e.g., 753.5 ng/L HR = 6.15; 95% CI 1.89–19.99; *p* = 0.003) (Figure 1).

### 3.7. Kaplan–Meier Models

**Figure 2:**
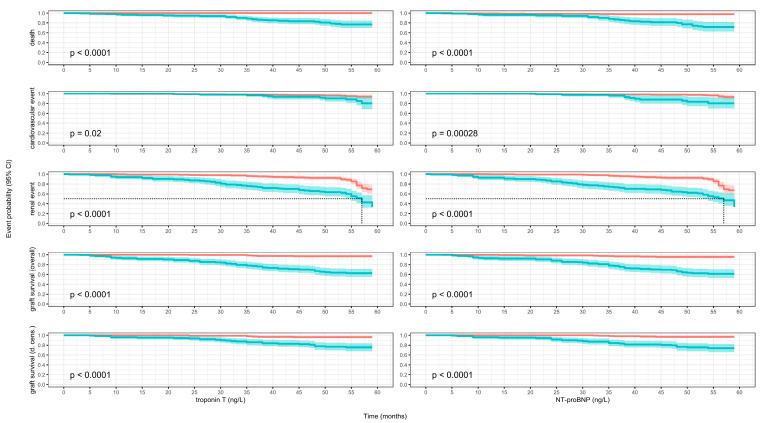
Kaplan–Meier survival curve estimates with 95% confidence intervals and log-rank test results. Red bold line: survival probability for patients with lower substance concentrations; blue bold line: survival probability for patients with higher substance concentrations; red and blue ribbons: 95% confidence intervals (Figure 2).

Death: Troponin T below cutoff: 0% (95% CI 0–0%) vs. above cutoff: 23% (95% CI 15–30%), representing a new event occurrence in nearly one in four patients with higher levels. NT-proBNP below cutoff: 3% (95% CI 1–5%) vs. above: 29% (95% CI 18–39%), a nearly tenfold relative increase (Figure 2).

Cardiovascular events: Troponin T: 6% (95% CI 2–10%) vs. 20% (95% CI 6–32%), more than tripling the event probability. NT-proBNP: 7% (95% CI 3–12%) vs. 21% (95% CI 8–31%), also a threefold relative rise (Figure 2).

Renal events: Troponin T: 31% (95% CI 21–39%) vs. 67% (95% CI 47–79%), more than doubling the risk. NT-proBNP: 33% (95% CI 23–41%) vs. 67% (95% CI 43–81%), with a similar doubling pattern (Figure 2).

Graft loss (overall survival): Troponin T: 3% (95% CI 1–6%) vs. 38% (95% CI 29–45%), more than a twelvefold increase. NT-proBNP: 4% (95% CI 2–7%) vs. 40% (95% CI 30–48%), a tenfold rise (Figure 2).

Graft loss (death-censored): Troponin T: 4% (95% CI 1–6%) vs. 25% (95% CI 16–33%), over six times higher. NT-proBNP: 3% (95% CI 1–5%) vs. 26% (95% CI 17–34%), nearly ninefold higher (Figure 2).

For renal events, only the high-concentration groups reached median event times of 57 months for both troponin T and NT-proBNP.

### 3.8. Time-Dependent Discrimination

Time-dependent ROC analysis indicated that several cutoffs retained good discriminative ability across follow-up.

For death, cTnT ≥ 12.25 ng/L yielded AUCs of 78.0% (95% CI 75.3–80.6) at 12 months, increasing to 81.8% (95% CI 78.8–84.7) at 48 months. For NT-proBNP ≥ 471.53 ng/L, corresponding AUCs were 74.3% (95% CI 56.6–91.9) and 77.5% (95% CI 68.8–86.2) at the same time points (Table 4).

For cardiovascular events, AUCs for the troponin T cutoff (15.63 ng/L) rose from 33.7% (95% CI 31.2–36.3) at 12 months to 60.5% (95% CI 46.6–74.4) at 48 months. NT-proBNP (673.94 ng/L) ranged from 37.1% (95% CI 34.7–39.5) to 72.9% (95% CI 59.5–86.2) (Table 4).

For renal events, AUCs for troponin T (15.68 ng/L) ranged from 77.7% (95% CI 65.9–89.5) at 12 months to 72.2% (95% CI 65.3–79.1) at 48 months. NT-proBNP (298.50 ng/L) started higher—84.4% (95% CI 81.9–86.9)—but declined to 73.1% (95% CI 66.3–80.0) (Table 4).

For overall graft survival, cTnT (13.37 ng/L) yielded AUCs of 80.3% (95% CI 77.7–83.0) at 12 months and 77.5% (95% CI 71.8–83.1) at 48 months. NT-proBNP (410.81 ng/L) ranged from 77.0% (95% CI 67.4–86.7) to 76.2% (95% CI 69.9–82.5) (Table 4).

For death-censored graft survival, cTnT (14.36 ng/L) ranged from 81.6% (95% CI 78.9–84.2) to 73.1% (95% CI 64.7–81.5). NT-proBNP (420.93 ng/L) ranged from 82.3% (95% CI 79.7–84.9) to 75.5% (95% CI 67.5–83.5) (Table 4).

Across endpoints, discrimination tended to be highest at earlier follow-up and modestly declined thereafter, though several cutoffs maintained AUCs > 0.75 even at 48 months.

## 4. Discussion

Our study aimed to further define the role of cTnT and NT-proBNP as prognostic biomarkers for all-cause mortality, cardiovascular, and renal events in real-life, stable RTRs. Using Cox regression, we identified cutoff values for both biomarkers and observed outcome associations at both the lowest and highest baseline concentrations. During the 4.9-year follow-up, the probability of death for patients with biomarker levels above the cutoff was 23% for cTnT and 29% for NT-proBNP, compared with 0% and 3%, respectively, for those below the cutoff. The probability of cardiovascular events was 20% for cTnT and 21% for NT-proBNP, compared with 6% and 7% in the lower-level groups. Renal event probability exceeded 65% for both biomarkers in the higher-level groups, compared with about one-third in the lower-level groups. Moreover, the probability of returning to dialysis or death (overall graft survival) was 38% for cTnT and 40% for NT-proBNP, versus 3–4% in their respective low-level groups. For death-censored graft survival, the probability was 25% for cTnT and 26% for NT-proBNP, compared with 4% and 3% in the low-concentration groups. Time-dependent ROC analysis showed that several cutoffs maintained good discrimination (AUC > 0.75) at early and intermediate follow-up, with some—particularly for renal outcomes and death-censored graft survival—remaining above this threshold even at 48 months. These findings suggest that assessment of both biomarkers—already in routine clinical use—may be valuable for identifying patients at risk of adverse outcomes, including graft survival, even several years after kidney transplantation.

In patients on the waiting list for kidney transplantation, elevated cTnT is a significant predictor of death, with a risk gradient observed for those with higher cTnT levels [18]. Survival following renal transplantation is predicted by pretransplant cTnT concentrations, with a higher risk in individuals with pre-existing CVD or cardiac risk factors. Additionally, 10-year mortality in this population was predicted by after kidney transplantation cTnT levels, with measurements taken 760 days after transplantation being associated with higher mortality, regardless of age, diabetes, pretransplant dialysis, cardiac illness, or allograft function [22].

The notion that therapies improving cardiac health, as shown by decreased cTnT levels, may lead to improved survival was supported by the substantial correlation between cTnT levels 1 year after kidney transplantation and survival in RTRs [15]. Avoiding or minimizing dialysis before transplantation is one of the potential targets for preventive or curative interventions suggested by the identification of characteristics associated with elevated cTnT [23,24].

It is well established that preemptive transplantation is advantageous, and that the duration of dialysis before transplantation is correlated with posttransplant survival. Additionally, we observed lower levels of cTnT and NT-proBNP in patients who received pre-emptive transplants and in those who underwent shorter periods of dialysis before transplantation. In contrast to the cited results, we assessed the biomarkers in the later period (7.5 years) after kidney transplantation, providing further evidence of their role in assessing the risk of death, CV, and renal events after kidney transplantation.

Our findings further indicate a correlation between cTnT levels and kidney transplant function. This finding aligns with existing research that highlights the relationship between allograft function and survival after transplantation. The alterations in cTnT levels may also result from CKD patients’ impaired ability to degrade the cTnT molecule normally due to disturbances in the clearance mechanism. Nevertheless, a decrease in GFR alone is insufficient to elevate cTnT levels [18]. Consequently, it is hypothesized that both decreased cTnT clearance and cardiac damage contribute to the elevated cTnT levels observed in certain CKD patients. This hypothesis is consistent with findings that patients with high CV risk—such as those with diabetes, atherosclerotic renal disease, and CKD—exhibit selectively increased cTnT levels [23,25,26].

The interest in utilizing allograft function as an outcome in transplantation therapy trials has been emphasized once again. While there are numerous interpretive challenges associated with this endpoint, measuring cTnT provides insight into the potential effects of decreased GFR on CV risk and could serve as a valuable metric for evaluating the efficacy of interventions aimed at improving graft function [27,28].

When left ventricular wall stress increases, which can occur in cardiac, renal, or hepatic failure, ventricular myocytes primarily release NPs. International recommendations support NT-proBNP as a classical biomarker for the diagnosis, prognosis, and therapeutic monitoring of patients with CVD, particularly those with HF, based on substantial research conducted on adults [29,30]. In patients with CKD, even those without HF, a decrease in GFR leads to an increase in NPs. Elevated BNP levels may result from increased cardiac release due to blood volume expansion, blood pressure elevation, and cardiac hypertrophy, but also partly from impaired renal clearance. In adult CKD patients, high levels of NPs are strong predictors of CV events and all-cause mortality. Conversely, patients with lower NPs during follow-up exhibit a better prognosis than those with elevated BNP [31].

Wei et al. showed that allograft renal transplantation results in a significant reduction in BNP starting on the first postoperative day. They observed a substantial rebound in BNP levels when allograft dysfunction occurred. In some patients with acute allograft dysfunction, changes in plasma BNP levels appeared several days before changes in blood creatinine. As a result, the authors suggested that plasma BNP could serve as a sensitive biomarker for the clinical detection of allograft dysfunction after kidney transplantation [32]. Moreover, NT-proBNP levels measured 2 or 3 weeks posttransplant by Bodlaj et al. were significantly correlated with the eGFR rate 1 year after transplantation [33]. In the present study, we assessed NT-proBNP during the late period after kidney transplantation and found that this biomarker is associated with the doubling of creatinine or graft survival, defined as renal events. Therefore, for the first time, we provide evidence that this biomarker is useful in evaluating the probability of renal events in the analyzed population.

Among stable RTRs enrolled in a homocysteine reduction clinical trial, Jarolim et al. found a statistically significant correlation between higher BNP and cTnI concentrations and clinical outcomes such as death, CV events, and dialysis-dependent kidney transplant failure. Interestingly, BNP showed a stronger correlation with clinical outcomes than cTnI. Higher BNP quartiles were associated with significantly increased risk ratios for all outcomes, both fatal and nonfatal. In contrast, the results did not show the same substantial correlations with cTnI quartiles as with BNP [17]. In contrast to these data, we assessed molecules such as cTnT and NT-proBNP at baseline, with the transplant vintage and follow-up time being longer in our population than in the aforementioned study. Finally, we showed the significant role of these biomarkers in predicting all-cause mortality, CV, and renal events in RTRs in real-life observations.

The prospective Heterogeneity of Monocytes and Echocardiography Among Allograft Recipients in Nephrology (HOME ALONE) study performed by Emrich et al. followed 177 RTRs for 5.4 ± 1.7 years. At baseline, the patients were 6.9 years after kidney transplantation, with graft function comparable to that of our study population. Additionally, plasma NT-proBNP and cTnT levels were analyzed. The authors established predefined outcomes, such as severe atherosclerotic CV events, all-cause mortality, and hospitalization for acute decompensated heart failure or all-cause death (HF/D). In conclusion, they demonstrated that cTnT and plasma NT-proBNP were independent predictors of these outcomes [34]. These data align with our results regarding the risk of death and CV events. However, we conducted our study in a larger RTR population (342 patients) and explored the role of biomarkers as predictors of renal events, which was not analyzed by Emrich et al. [34].

Finally, in the liver transplantation (LT) population, Kwon et al. assessed the relationship between the risk of postLT mortality and pretransplant BNP levels and/or peak BNP levels within the first three postoperative days (postBNPPOD3) by using serial testing to assess changes in BNP levels before and after transplantation. Specifically, a BNP level > 400 pg/mL is recognized as the cutoff for acute HF, and it may serve as an early indicator of HF or suggest the likelihood of overt HF. Regardless of the presence of HF signs and symptoms, the authors of this study demonstrated an association between postBNPPOD3 levels > 400 pg/mL and postLT mortality [35].

### 4.1. Limitations

A few limitations of our studies warrant discussion. First, patients in our transplant program were all from a single transplant center. Moreover, we did not conduct a separate analysis based on the type of donor (living or deceased). However, these factors do not affect the results of our analyses, as the relationship between cTnT or NT-proBNP and survival, CV events, and renal events remained significant across the total cohort of RTRs. Second, the biomarkers were measured only once at baseline and SGLT2 (sodium-glucose cotransporter 2) inhibitors were not used. Finally, since these data are observational, we can only speculate about the mechanisms underlying the observed relationships. Nonetheless, these results provide a valuable baseline for future studies that use cTnT or NT-proBNP as targets for intervention.

### 4.2. Pro

It is important to emphasize that we obtained complete data for all participants regarding the primary and secondary endpoints after the follow-up. Moreover, this is the first study to assess the role of cTnT and NT-proBNP in identifying the risk of death, CV and renal events, and overall graft survival during the late period after kidney transplantation, with the mean time in our study population being 93 months.

## 5. Conclusions

Biomarkers such as cTnT and NT-proBNP appear promising in identifying patients at high risk of death, CV and renal events, and overall graft failure. Our data may be valuable for future research on cardioprotective and nephroprotective interventions in RTRs during the late period after kidney transplantation.

## Figures and Tables

**Table 1:** Baseline characteristics by cTnT quartiles (ng/L).

Characteristic	Overall, *N* = 342 ^a^	[0, 7], *N* = 108 ^a^	[7, 11.5], *N* = 63 ^a^	[11.5, 21], *N* = 86 ^a^	[21, 48.9], *N* = 85 ^a^	*p*-Value ^b^
Age (years)	53 (14)	45 (12)	51 (14)	55 (13)	62 (11)	<0.001
Sex	-	-	-	-	-	<0.001
Female	129/342 (38%)	59/108 (55%)	22/63 (35%)	28/86 (33%)	20/85 (24%)	
Male	213/342 (62%)	49/108 (45%)	41/63 (65%)	58/86 (67%)	65/85 (76%)	
BMI (kg/m^2^)	25.6 (4.8)	25.0 (4.7)	25.9 (5.0)	25.4 (4.6)	26.4 (4.8)	0.086
Total weight (kg)	76 (17)	73 (17)	77 (16)	76 (17)	81 (16)	0.010
DM type 2 (yes)	45/342 (13%)	7/108 (6.5%)	10/63 (16%)	10/86 (12%)	18/85 (21%)	0.023
NODAT (yes)	21/342 (6.1%)	7/108 (6.5%)	3/63 (4.8%)	6/86 (7.0%)	5/85 (5.9%)	>0.9
CAD (yes)	77/342 (23%)	2/108 (1.9%)	9/63 (14%)	16/86 (19%)	50/85 (59%)	<0.001
Heart failure (yes)	90/342 (26%)	19/108 (18%)	11/63 (17%)	26/86 (30%)	34/85 (40%)	0.001
Hypertension (yes)	296/342 (87%)	92/108 (85%)	53/63 (84%)	76/86 (88%)	75/85 (88%)	0.8
Glomerulonephritis (yes)	185/342 (54%)	67/108 (62%)	31/63 (49%)	50/86 (58%)	37/85 (44%)	0.052
Polycystic kidney disease (yes)	55/342 (16%)	12/108 (11%)	11/63 (17%)	14/86 (16%)	18/85 (21%)	0.3
Tubulointerstitial nephritis (yes)	69/342 (20%)	27/108 (25%)	12/63 (19%)	12/86 (14%)	18/85 (21%)	0.3
Hypertensive nephropathy (yes)	18/342 (5.3%)	1/108 (0.9%)	2/63 (3.2%)	6/86 (7.0%)	9/85 (11%)	0.013
Unknown etiology (yes)	10/342 (2.9%)	1/108 (0.9%)	7/63 (11%)	2/86 (2.3%)	0/85 (0%)	<0.001
Time of RRT (months)	59 (61)	36 (53)	67 (76)	74 (60)	68 (51)	<0.001
Time post KTx (months)	93 (80)	100 (78)	79 (83)	89 (77)	98 (81)	0.13
Preemptive KTX (yes) n (%)	47/342 (14%)	27/108 (25%)	9/63 (14%)	4/86 (4.7%)	7/85 (8.2%)	<0.001
Creatinine (mg/dL)	1.67 (0.81)	1.29 (0.37)	1.51 (0.51)	1.69 (0.76)	2.27 (1.06)	<0.001
eGFR CKDEPI (mL/min/1.73 m^2^)	50 (20)	62 (17)	54 (18)	48 (18)	35 (17)	<0.001
NT pro BNP (ng/L)	684 (934)	231 (357)	411 (661)	673 (816)	1473 (1196)	<0.001
Cyclosporine (yes)	72/342 (21%)	24/108 (22%)	11/63 (17%)	17/86 (20%)	20/85 (24%)	0.8
Tacrolimus (yes)	212/342 (62%)	75/108 (69%)	43/63 (68%)	57/86 (66%)	37/85 (44%)	<0.001
Steroids (yes)	176/342 (51%)	38/108 (35%)	37/63 (59%)	45/86 (52%)	56/85 (66%)	<0.001
MMF (yes)	150/342 (44%)	45/108 (42%)	23/63 (37%)	44/86 (51%)	38/85 (45%)	0.3
MPS (yes)	170/342 (50%)	59/108 (55%)	35/63 (56%)	40/86 (47%)	36/85 (42%)	0.3
Betalacept (yes)	41/342 (12%)	8/108 (7.4%)	6/63 (9.5%)	9/86 (10%)	18/85 (21%)	0.023
Calcium channel blocker (yes)	157/342 (46%)	37/108 (34%)	29/63 (46%)	48/86 (56%)	43/85 (51%)	0.018
ACE inhibitor (yes)	89/342 (26%)	25/108 (23%)	16/63 (25%)	27/86 (31%)	21/85 (25%)	0.6
Statines (yes)	142/342 (42%)	34/108 (31%)	22/63 (35%)	44/86 (51%)	42/85 (49%)	0.011
AT1-R antagonists (yes)	111/342 (32%)	28/108 (26%)	17/63 (27%)	31/86 (36%)	35/85 (41%)	0.093

^a^ Mean (SD); *n*/*N* (%). ^b^ One-way ANOVA; Pearson’s Chi-squared test; Kruskal–Wallis rank sum test; Fisher’s exact test. SI conversion factor: for creatinine to convert mg/dL to µmol/L, multiply by 88.42. Abbreviations: BMI—body mass index, DM—diabetes mellitus, NODAT—new onset diabetes after transplantation, CAD—coronary artery disease, RRT—renal replacement therapy, Ktx—kidney transplantation, eGFR—estimated glomerular filtration rate, NT-proBNP—N-terminal pro-B-type natriuretic peptide, ACE—angiotensin converting enzyme, AT1-R—angiotensin receptor 1.

**Table 2:** Baseline characteristics by NT-proBNP quartiles (pg/mL).

Characteristic	Overall, *N* = 342 ^a^	[0, 113], *N* = 87 ^a^	[113, 298], *N* = 84 ^a^	[298, 754], *N* = 85 ^a^	[754, 3376], *N* = 86 ^a^	*p*-Value ^b^
Age	53 (14)	46 (12)	50 (13)	56 (13)	58 (14)	<0.001
Sex						0.008
Female	129/342 (38%)	20/87 (23%)	33/84 (39%)	40/85 (47%)	36/86 (42%)	
Male	213/342 (62%)	67/87 (77%)	51/84 (61%)	45/85 (53%)	50/86 (58%)	
BMI (kg/m^2^)	25.6 (4.8)	25.8 (4.2)	25.9 (5.0)	25.1 (4.7)	25.6 (5.3)	0.6
Total weight (kg)	76 (17)	79 (16)	77 (17)	74 (16)	75 (18)	0.2
DM type 2 (yes)	45/342 (13%)	11/87 (13%)	9/84 (11%)	9/85 (11%)	16/86 (19%)	0.4
NODAT (yes)	21/342 (6.1%)	8/87 (9.2%)	5/84 (6.0%)	3/85 (3.5%)	5/86 (5.8%)	0.5
CAD (yes)	77/342 (23%)	3/87 (3.4%)	9/84 (11%)	22/85 (26%)	43/86 (50%)	<0.001
Heart failure (yes)	90/342 (26%)	17/87 (20%)	12/84 (14%)	26/85 (31%)	35/86 (41%)	<0.001
Hypertension (yes)	296/342 (87%)	78/87 (90%)	69/84 (82%)	71/85 (84%)	78/86 (91%)	0.3
Glomrulonephritis (yes)	185/342 (54%)	48/87 (55%)	55/84 (65%)	36/85 (42%)	46/86 (53%)	0.027
Polycystic kidney disease (yes)	55/342 (16%)	11/87 (13%)	14/84 (17%)	20/85 (24%)	10/86 (12%)	0.14
Tubulointerstitial nephritis (yes)	69/342 (20%)	23/87 (26%)	10/84 (12%)	17/85 (20%)	19/86 (22%)	0.12
Hypertensive nephropathy (yes)	18/342 (5.3%)	2/87 (2.3%)	2/84 (2.4%)	7/85 (8.2%)	7/86 (8.1%)	0.12
Unknown etiology (yes)	10/342 (2.9%)	1/87 (1.1%)	3/84 (3.6%)	6/85 (7.1%)	0/86 (0%)	0.020
Time of RRT (months)	59 (61)	46 (69)	59 (64)	62 (54)	71 (52)	<0.001
Time post KTx (months)	93 (80)	97 (85)	86 (77)	84 (73)	103 (82)	0.4
Preempitve KTX (yes)	47/342 (14%)	23/87 (26%)	12/84 (14%)	8/85 (9.4%)	4/86 (4.7%)	<0.001
Creatinine (mg/dL)	1.67 (0.81)	1.38 (0.38)	1.40 (0.44)	1.60 (0.63)	2.31 (1.14)	<0.001
eGFR CKDEPI (mL/min/1.73 m^2^)	50 (20)	61 (16)	57 (17)	48 (19)	34 (16)	<0.001
Troponin T (ng/L)	16 (13)	8 (5)	12 (10)	17 (12)	27 (14)	<0.001
Cyclosporine (yes)	72/342 (21%)	15/87 (17%)	17/84 (20%)	18/85 (21%)	22/86 (26%)	0.6
Tacrolimus (yes)	212/342 (62%)	61/87 (70%)	53/84 (63%)	54/85 (64%)	44/86 (51%)	0.077
Steroids (yes)	176/342 (51%)	31/87 (36%)	40/84 (48%)	49/85 (58%)	56/86 (65%)	<0.001
MMF (yes)	150/342 (44%)	44/87 (51%)	33/84 (39%)	34/85 (40%)	39/86 (45%)	0.4
MPS (yes)	170/342 (50%)	40/87 (46%)	45/84 (54%)	47/85 (55%)	38/86 (44%)	0.4
Betalacept (yes)	41/342 (12%)	8/87 (9.2%)	10/84 (12%)	9/85 (11%)	14/86 (16%)	0.5
Calcium channel blocker (yes)	157/342 (46%)	38/87 (44%)	38/84 (45%)	33/85 (39%)	48/86 (56%)	0.2
ACE inhibitor (yes)	89/342 (26%)	19/87 (22%)	21/84 (25%)	24/85 (28%)	25/86 (29%)	0.7
Statines (yes)	142/342 (42%)	35/87 (40%)	35/84 (42%)	29/85 (34%)	43/86 (50%)	0.2
AT1-R antagonists (yes)	111/342 (32%)	23/87 (26%)	24/84 (29%)	32/85 (38%)	32/86 (37%)	0.3

^a^ Mean (SD); *n*/*N* (%). ^b^ One-way ANOVA; Pearson’s Chi-squared test; Kruskal–Wallis rank sum test; Fisher’s exact test. SI conversion factor: for creatinine to convert mg/dL to µmol/L, multiply by 88.42. Abbreviations: BMI—body mass index, DM—diabetes mellitus, NODAT—new onset diabetes after transplantation, CAD—coronary artery disease, RRT—renal replacement therapy, Ktx—kidney transplantation, eGFR—estimated glomerular filtration rate, NT-proBNP—N-terminal pro-B-type natriuretic peptide, ACE—angiotensin converting enzyme, AT1-R—angiotensin receptor 1.

**Table 3:** Overall and conditional estimates of relative hazard (HR) for quartiles and estimated cutoff points.

Substance	Event	Quartile	Estimate1	*p*-Value1	Cut-Off	Estimate2	*p*-Value2
**Overall HR**							
troponin T (ng/L)	death	7	0.30 (0.07; 1.27)	0.10	12.25	0.98 (0.27; 3.50)	>0.9
		11.5	0.83 (0.24; 2.94)	0.8			
		21	4.11 (0.87; 19.38)	0.074			
		48.95	7.74 (1.56; 38.41)	0.012			
**Conditional HR**							
troponin T (ng/L)	death	[2.00, 7.00)	0.07 (0.01; 0.39)	0.003	[2.00, 12.25)	0.19 (0.05; 0.74)	0.017
		[7.00, 11.50)	0.36 (0.10; 1.35)	0.13			
		[11.50, 21.00)	2.05 (0.54; 7.79)	0.3	[12.25, 48.95]	6.98 (1.70; 28.72)	0.007
		[21.00, 48.95]	15.98 (3.45; 74.04)	<0.001			
**Overall HR**							
NT-proBNP (ng/L)	death	113	0.67 (0.21; 2.15)	0.5	471.53	1.36 (0.44; 4.19)	0.6
		298.5	0.97 (0.32; 2.91)	>0.9			
		753.5	2.31 (0.67; 7.95)	0.2			
		3376.05	7.43 (1.54; 35.83)	0.013			
**Conditional HR**							
NT-proBNP (ng/L)	death	[6.00, 113.00)	0.34 (0.09; 1.31)	0.12	[6.00, 471.53)	0.56 (0.17; 1.79)	0.3
		[113.00, 298.50)	0.58 (0.19; 1.77)	0.3			
		[298.50, 753.50)	1.56 (0.54; 4.56)	0.4	[471.53, 3376.05]	8.26 (2.51; 27.22)	<0.001
		[753.50, 3376.05]	11.45 (3.24; 40.48)	<0.001			
**Overall HR**							
troponin T (ng/L)	cardiovascular event	7	1.33 (0.30; 5.96)	0.7	15.63	1.41 (0.35; 5.72)	0.6
		11.5	1.37 (0.33; 5.77)	0.7			
		21	1.47 (0.37; 5.84)	0.6			
		48.95	1.78 (0.28; 11.31)	0.5			
**Conditional HR**							
troponin T (ng/L)	cardiovascular event	[2.00, 7.00)	0.62 (0.16; 2.45)	0.5	[2.00, 15.63)	0.91 (0.23; 3.62)	0.9
		[7.00, 11.50)	1.08 (0.25; 4.65)	>0.9			
		[11.50, 21.00)	1.62 (0.39; 6.67)	0.5	[15.63, 48.95]	3.30 (0.69; 15.69)	0.13
		[21.00, 48.95]	3.59 (0.73; 17.63)	0.11			
**Overall HR**							
NT-proBNP (ng/L)	cardiovascular event	113	1.06 (0.24; 4.63)	>0.9	673.94	1.61 (0.37; 6.95)	0.5
		298.5	1.22 (0.28; 5.28)	0.8			
		753.5	1.70 (0.39; 7.38)	0.5			
		3376.05	11.68 (1.73; 78.93)	0.012			
**Conditional HR**							
NT-proBNP (ng/L)	cardiovascular event	[6.00, 113.00)	0.80 (0.17; 3.71)	0.8	[6.00, 673.94)	1.03 (0.24; 4.35)	>0.9
		[113.00, 298.50)	1.03 (0.26; 4.17)	>0.9			
		[298.50, 753.50)	1.48 (0.35; 6.31)	0.6	[673.94, 3376.05]	5.24 (1.03; 26.77)	0.046
		[753.50, 3376.05]	5.57 (1.07; 29.01)	0.041			
**Overall HR**							
troponin T (ng/L)	renal event	7	3.15 (1.47; 6.76)	0.003	15.68	4.02 (1.98; 8.18)	<0.001
		11.5	3.58 (1.72; 7.43)	<0.001			
		21	4.67 (2.32; 9.37)	<0.001			
		48.95	10.21 (4.31; 24.17)	<0.001			
**Conditional HR**							
troponin T (ng/L)	renal event	[2.00, 7.00)	1.81 (0.90; 3.62)	0.094	[2.00, 15.68)	2.49 (1.23; 5.02)	0.011
		[7.00, 11.50)	2.80 (1.34; 5.85)	0.006			
		[11.50, 21.00)	3.93 (1.91; 8.10)	<0.001	[15.68, 48.95]	10.13 (4.66; 22.04)	<0.001
		[21.00, 48.95]	12.49 (5.65; 27.60)	<0.001			
**Overall HR**							
NT-proBNP (ng/L)	renal event	113	3.19 (1.52; 6.71)	0.002	471.53	4.35 (2.14; 8.84)	<0.001
		298.5	3.75 (1.85; 7.63)	<0.001			
		753.5	5.49 (2.61; 11.54)	<0.001			
		3376.05	8.07 (2.98; 21.83)	<0.001			
**Conditional HR**							
NT-proBNP (ng/L)	renal event	[6.00, 113.00)	1.99 (0.96; 4.13)	0.065	[6.00, 471.53)	2.62 (1.31; 5.25)	0.007
		[113.00, 298.50)	2.59 (1.31; 5.11)	0.006			
		[298.50, 753.50)	4.57 (2.26; 9.26)	<0.001	[471.53, 3376.05]	12.01 (5.54; 26.03)	<0.001
		[753.50, 3376.05]	15.12 (6.72; 34.01)	<0.001			
**Overall HR**							
troponin T (ng/L)	graft survival (overall)	7	0.64 (0.24; 1.67)	0.4	13.37	1.32 (0.55; 3.21)	0.5
		11.5	1.07 (0.45; 2.58)	0.9			
		21	2.81 (1.08; 7.31)	0.034			
		48.95	6.92 (2.52; 19.00)	<0.001			
**Conditional HR**							
troponin T (ng/L)	graft survival (overall)	[2.00, 7.00)	0.24 (0.09; 0.65)	0.005	[2.00, 13.37)	0.47 (0.19; 1.14)	0.10
		[7.00, 11.50)	0.66 (0.27; 1.62)	0.4			
		[11.50, 21.00)	1.60 (0.65; 3.94)	0.3	[13.37, 48.95]	5.85 (2.35; 14.56)	<0.001
		[21.00, 48.95]	11.12 (4.25; 29.09)	<0.001			
**Overall HR**							
NT-proBNP (ng/L)	graft survival (overall)	113	0.78 (0.32; 1.88)	0.6	410.81	1.91 (0.84; 4.37)	0.12
		298.5	1.38 (0.62; 3.08)	0.4			
		753.5	4.66 (1.80; 12.10)	0.002			
		3376.05	8.98 (2.61; 30.92)	<0.001			
**Conditional HR**							
NT-proBNP (ng/L)	graft survival (overall)	[6.00, 113.00)	0.45 (0.17; 1.18)	0.10	[6.00, 410.81)	0.66 (0.29; 1.55)	0.3
		[113.00, 298.50)	0.71 (0.32; 1.59)	0.4			
		[298.50, 753.50)	2.21 (0.98; 4.98)	0.055	[410.81, 3376.05]	11.18 (4.54; 27.51)	<0.001
		[753.50, 3376.05]	19.61 (7.36; 52.25)	<0.001			
**Overall HR**							
troponin T (ng/L)	graft survival (d. cens.)	7	1.27 (0.36; 4.41)	0.7	14.36	2.14 (0.69; 6.70)	0.2
		11.5	1.75 (0.55; 5.57)	0.3			
		21	3.34 (1.05; 10.62)	0.041			
		48.95	9.46 (2.70; 33.13)	<0.001			
**Conditional HR**							
troponin T (ng/L)	graft survival (d. cens.)	[2.00, 7.00)	0.54 (0.16; 1.85)	0.3	[2.00, 14.36)	0.96 (0.31; 3.02)	>0.9
		[7.00, 11.50)	1.22 (0.37; 4.00)	0.7			
		[11.50, 21.00)	2.08 (0.65; 6.64)	0.2	[14.36, 48.95]	7.64 (2.36; 24.72)	<0.001
		[21.00, 48.95]	13.87 (4.13; 46.55)	<0.001			
**Overall HR**							
NT-proBNP (ng/L)	graft survival (d. cens.)	113	1.56 (0.50; 4.83)	0.4	420.93	3.12 (1.09; 8.93)	0.034
		298.5	2.39 (0.85; 6.73)	0.10			
		753.5	6.15 (1.89; 19.99)	0.003			
		3376.05	10.25 (2.31; 45.51)	0.002			
**Conditional HR**							
NT-proBNP (ng/L)	graft survival (d. cens.)	[6.00, 113.00)	0.80 (0.24; 2.65)	0.7	[6.00, 420.93)	1.17 (0.40; 3.43)	0.8
		[113.00, 298.50)	1.20 (0.43; 3.35)	0.7			
		[298.50, 753.50)	3.58 (1.26; 10.19)	0.017	[420.93, 3376.05]	16.94 (5.35; 53.68)	<0.001
		[753.50, 3376.05]	28.52 (8.21; 99.04)	<0.001			

The analysis was adjusted for age, sex, coronary artery disease (CAD), the value of estimated glomerular filtration rate (eGFR), administration of tacrolimus, cyclosporine, steroids and time after kidney transplantation. Abbreviations: CAD—coronary artery disease, HR—hazard ratio, NT-proBNP—N-terminal pro-B-type natriuretic peptide, d.cens.—death censored.

**Table 4:** Time-dependent area under the ROC curve (AUC) for troponin T and NT-proBNP cutoffs in predicting outcomes at 12, 24, 36, and 48 months. Values are given as percentages with 95% confidence intervals.

Substance	Event	Month	AUC	95% CI
troponin T (ng/L)	death	12.00	77.96	75.28; 80.65
		24.00	78.57	75.8; 81.34
		36.00	80.04	77.18; 82.89
		48.00	81.76	78.81; 84.72
NT-proBNP (ng/L)		12.00	74.26	56.61; 91.91
		24.00	65.96	48.95; 82.96
		36.00	73.96	63.14; 84.77
		48.00	77.46	68.76; 86.15
troponin T (ng/L)	cardiovascular event	12.00	33.74	31.2; 36.29
		24.00	46.52	25.08; 67.97
		36.00	57.10	38.45; 75.74
		48.00	60.52	46.64; 74.4
NT-proBNP (ng/L)		12.00	37.12	34.74; 39.49
		24.00	50.15	28.73; 71.58
		36.00	60.86	42.25; 79.47
		48.00	72.86	59.54; 86.17
troponin T (ng/L)	renal event	12.00	77.66	65.85; 89.48
		24.00	75.23	65.26; 85.2
		36.00	74.14	66.48; 81.8
		48.00	72.17	65.29; 79.05
NT-proBNP (ng/L)		12.00	84.41	81.89; 86.94
		24.00	79.08	70.57; 87.6
		36.00	76.47	69.12; 83.83
		48.00	73.12	66.25; 79.98
troponin T (ng/L)	graft survival (overall)	12.00	80.34	77.69; 82.98
		24.00	80.94	78.22; 83.66
		36.00	77.43	71.68; 83.18
		48.00	77.48	71.83; 83.13
NT-proBNP (ng/L)		12.00	77.02	67.36; 86.67
		24.00	72.39	61.9; 82.89
		36.00	74.77	67.63; 81.91
		48.00	76.17	69.87; 82.47
troponin T (ng/L)	graft survival (d. cens.)	12.00	81.55	78.94; 84.17
		24.00	82.08	79.4; 84.77
		36.00	73.58	64.59; 82.58
		48.00	73.06	64.67; 81.46
NT-proBNP (ng/L)		12.00	82.32	79.73; 84.91
		24.00	82.74	80.07; 85.4
		36.00	76.85	68.69; 85.01
		48.00	75.50	67.46; 83.54

Abbreviation: NT-proBNP—N-terminal pro-B-type natriuretic peptide.

## Data Availability

The raw data supporting the conclusions of this article will be made available by the authors on request.

## Abbreviations

The following abbreviations are used in this manuscript:

RTRs	Renal transplant recipients
cTnT	Cardiac Troponin T
NTproBNP	N-terminal pro-B-type natriuretic peptide
cTnI	Cardiac Troponin I
CVD	Cardio-vascular disease
CKD	Chronic kidney disease
BNP	B-type natriuretic peptide
HR	Hazard ratio
eGFR	Estimated glomerular filtration rate
HF	Heart failure
NPs	Natriuretic peptides
